# La sobreexpresión de FoxO1 en el hígado esta positivamente asociada al grado de daño hepático en pacientes cirróticos

**DOI:** 10.1515/almed-2023-0054

**Published:** 2023-08-04

**Authors:** Esther Fernández-Galán, Silvia Sandalinas, Laura Macias-Muñoz, Irene Portolés, Jordi Ribera, Blai Morales-Romero, Montse Pauta, Gregori Casals, Loreto Boix, Wladimiro Jiménez, Manuel Morales-Ruiz

**Affiliations:** Servicio de Bioquímica y Genética Molecular, Centro de Diagnóstico Biomédico (CDB), Hospital Clinic de Barcelona, Barcelona, España; Institut d’Investigacions Biomèdiques August Pi i Sunyer (IDIBAPS), Centro de Investigación Biomédica en Red de Enfermedades Hepáticas y Digestivas (CIBERehd), Barcelona, España; Comisión para la Valoración Bioquímica de la Enfermedad Hepática-SEQC^ML^ , Barcelona, España; Barcelona Clinic Liver Cancer Group, Unidad de Hepatología, Hospital Clínic de Barcelona, Barcelona, España; Servicio de Biomedicina-Unidad de Bioquímica, Facultad de Medicina y Ciencias de la Salud, Universidad de Barcelona, Barcelona, España

**Keywords:** Akt, cirrosis hepática, enfermedad hepática crónica, FoxO1, regeneración hepática

## Abstract

**Objetivos:**

La enfermedad hepática crónica y sus complicaciones, la cirrosis y el carcinoma hepatocelular, presentan una elevada mortalidad. Los tratamientos curativos, como la hepatectomía parcial o el trasplante hepático, tienen una aplicación limitada en pacientes con cirrosis, por su escasa capacidad de regeneración hepática. Son necesarias otras alternativas diagnósticas y terapéuticas para prevenir la progresión de la enfermedad y mejorar la supervivencia. Diversos estudios preclínicos demuestran el importante papel de la proteína quinasa B(Akt) en la disfunción hepática, aunque aún se desconoce el estado de Akt y sus dianas en las patologías hepáticas crónicas. El principal objetivo es determinar el estado de activación de la vía Akt y su relación con la función hepática en pacientes cirróticos.

**Métodos:**

Estudio retrospectivo con muestras de tejido hepático de 36 pacientes hepatectomizados con (n=27) y sin (n=9) cirrosis. Se realizó un análisis Multiplex de las proteínas de la vía Akt/mTOR empleando un panel Luminex y *Western blot*. Previamente a la resección, se realizaron las pruebas habituales de función hepática en suero.

**Resultados:**

Akt y la proteína FoxO1 están sobreexpresadas en el hígado cirrótico: 1.0 unidades densitométricas relativas (UDR); p<0,01, y 9,5 vs*.* 4,4 DRU; p<0,01, respectivamente). FoxO1 mostró una fuerte correlación con los marcadores de daño hepático (aspartato aminotransferasa (ASAT): r=0,51, p<0,05; alanina aminotransferasa (ALAT): r=0,49, p<0,05), y fue la única enzima de la vía Akt identificada como predictor independiente de los niveles de ASAT y ALAT.

**Conclusiones:**

La expresión intrahepática de FoxO1 podría tener utilidad clínica como marcador pronóstico en la enfermedad hepática avanzada.

## Introducción

Las patologías hepáticas requieren frecuentes visitas de seguimiento, debido a la complejidad de su manejo clínico, estando asociadas a frecuentes hospitalizaciones y elevadas tasas de mortalidad, por complicaciones como el carcinoma hepatocelular (HCC) y la cirrosis. Estas patologías son las segundas en haber experimentado un mayor crecimiento en los últimos cinco años [1]. Cabe destacar que esta enfermedad afecta a la población activa, lo que tiene un impacto sobre la productividad de la sociedad. El trasplante hepático es el tratamiento definitivo para la enfermedad hepática terminal, mejorando la supervivencia y calidad de vida. Sin embargo, el rechazo crónico del injerto, la limitada capacidad de regeneración del hígado de estos pacientes, así como el desequilibrio entre la demanda y la disponibilidad de órganos, compromete la eficacia de esta estrategia terapéutica.

Es perentorio hallar nuevas alternativas diagnósticas y terapéuticas para estos pacientes, destinadas a bloquear la progresión de la enfermedad y prevenir la insuficiencia orgánica y sus comorbilidades, así como reducir los costes socioeconómicos.

Diversos estudios demuestran que la proteína kinasa b (Akt) desempeña un papel crucial en la regulación de la función hepática. En modelos de ratón, la cirrosis hepática se ha relacionado con una menor actividad de Akt, mientras que, al restablecer esta función mediante terapia génica, la hemodinámica hepática mejoró [2]. Diversos mecanismos contribuyen a la desregulación de Akt en la disfunción hepática. Se ha hallado evidencia de la relación entre el receptor acoplado a proteínas G (GRK2), un inhibidor de la señalización del receptor acoplado a la proteína G, y la activación del óxido nítrico sintasa endotelial (eNOS) mediado por Akt, en modelos de fibrosis hepática [3].

Un estudio en modelos de trasplante hepático ortotópico en cerdos confirmó que la activación de la vía Akt tenía un efecto beneficioso sobre la función hepática. Así, el daño por isquemia-reperfusión durante el trasplante fue la principal causa de estrés oxidativo y muerte celular, estando este asociado clínicamente a la pérdida de funcionalidad, así como a un mayor riesgo de rechazo del injerto. Mediante la activación constitutiva de la vía Akt a través de la expresión forzada de una variante de Akt (myrAKT) en injertos hepáticos transducidos, se restableció la función hepática y se contrarrestaron los procesos apoptóticos, aumentando la viabilidad del injerto [4]. Esto concuerda con estudios anteriores, que señalan la importancia de la vía Akt en el mantenimiento de los principales procesos fisiológicos del hígado.

Estudios recientes también sugieren que la vía Akt ejerce un papel fundamental en la regeneración hepática. También existe evidencia de que, durante la regeneración, Akt es fosforilada (activa), previniendo la apóptosis de los hepatocitos, al inducir la activación de las vías de señalización de supervivencia celular [5, 6]. La deficiencia de la proteína Akt o de sus moléculas de activación, la proteína quinasa 1 dependiente de la proteína 3-fosfoinosítidos (PDK1) y las fosfoinosítido-3-cinasas (PI3-K), reduce la capacidad de regeneración hepática, lo que conlleva una mayor mortalidad tras la hepapectomía parcial [7]. Esto se explica por la acción de Akt y de algunas de sus dianas o activadores como reguladores positivos de la supervivencia celular, como el receptor de la insulina (RI), el sustrato del receptor de insulina 1; y el Fosfatidilinositol 3,4,5-trifosfato 3-fosfatasa (PTEN).

La Akt es un mediador crucial del crecimiento celular, a través de la regulación directa de la diana de la rapamicina en los mamíferos (mTOR). Un estudio llevado a cabo con modelos de hepatectomía parcial en ratones con deficiencia de la proteína FoxO1, demostró que la inhibición específica de FoxO1 en los hepatocitos mediada por Akt es esencial para la regeneración hepática [8].

A pesar de la evidencia de que la actividad de Akt influye en la fisiología y patofisiología del hígado, la mayoría de los estudios se han realizado en modelos experimentales de roedores. Así, sigue desconociéndose el estado de activación de Akt y de sus procesos moleculares diana posteriores *(downstream targets)* en patologías hepáticas crónicas. Esta falta de información limita la transferencia de los conocimientos adquiridos en los estudios preclínicos al desarrollo de nuevas terapias y biomarcadores en clínica. Por este motivo, la evaluación del estado de activación de Akt y sus dianas moleculares en pacientes con enfermedad hepática crónica es clínicamente relevante.

El objetivo del presente estudio era evaluar el estado de activación de las proteínas implicadas en la vía Akt/mTOR en el hígado de pacientes con cirrosis. Así mismo, se analizó la asociación entre las proteínas Akt y el daño y la disfunción hepática, y se determinó su valor diagnóstico y pronóstico.

## Materiales y métodos

### Diseño y sujetos de estudio

En el presente estudio retrospectivo, se incluyeron muestras de pacientes con y sin cirrosis (confirmada mediante biopsia), sometidos a una hepatectomía parcial terapéutica. Se analizaron 36 tejidos hepáticos, de los cuales 27 eran tejidos cirróticos, siendo los otros nueve tejidos sanos (grupo no cirrótico). Las muestras de hígado cirrótico se obtuvieron mediante resección hepática, en pacientes con cirrosis secundaria a infección por el virus de la hepatitis C (VHC). Las muestras del “grupo no cirrótico” contenían tejido hepático sano, obtenido durante la resección de metástasis colorrectales previa al clampaje. Las muestras con presencia histológica de tejido tumoral se excluyeron de posteriores análisis. En la Tabla 1 se muestran las características demográficas de los pacientes (edad y sexo).

**Tabla 1:** Características demográficas de los pacientes incluidos en el estudio.

Grupo	No cirróticos (n=9)	Cirróticos (n=27)	Total	Valor p
Edad, años mediana (RIC)	51,0 (36,0–66,0)	66,5 (57,0–69,0)	65,5 (51,5–68,5)	NE
Sexo % masculino	44,4 %	84,6 %	74,3 %	0,03

RIC, rango intercuartílico.

### Parámetros analíticos de daño y función hepática

En los pacientes cirróticos, se evaluaron diversos biomarcadores de daño y función hepática, para estudiar su posible correlación con las proteínas diana de la vía Akt. Para tal fin, se midieron los siguientes marcadores bioquímicos: aspartato aminotransferasa (ASAT), alanina aminotransferasa (ALAT), gamma-glutamil transferasa (GGT), albúmina y bilirrubina. También se midieron los niveles del marcador tumoral alfafetoproteína (AFP) y otros parámetros incluidos en un panel de marcadores hepáticos: glucosa, colesterol y triglicéridos. Los parámetros fueron determinados en suero previamente a la resección quirúrgica. Todos los análisis se realizaron en el laboratorio central del Hospital Clínic de Barcelona, en los analizadores automáticos ADVIA 2400 Chemistry System y ADVIA Centaur XP (Siemens Healthineers, NY, EE.UU). La puntuación FIB-4, utilizada para calcular la extensión de la fibrosis hepática en pacientes con hepatitis crónica o cirrosis, se calculó en función de cuatro variables: edad, niveles séricos de ASAT y ALAT, y recuento de plaquetas en sangre [9].

### Análisis de proteínas de la vía Akt/mTOR

Se determinaron los niveles de fosfoproteínas 10 de la vía de señalización Akt/mTOR en 36 muestras de tejido hepático. A continuación, se enumeraron los analitos cuantificados mediante inmunoensayo multiplex xMAP^®^ de Luminex^®^, un ensayo con placas de 96 pocillos sobre 11 proteínas con un panel de microbolas magnéticas de fosfoproteínas de la vía Akt/mTOR (Merck Millipore, Darmstadt, Alemania): insulina (IGF1R); sustrato del receptor de insulina 1 (IRS1); mTOR; proteína cinasa S6 ribosomal beta-1 (p70S6K); RI; PTEN; glucógeno sintasa quinasa 3 alfa (GSK3α); complejo de esclerosis tuberosa 2 (TSC2); y proteína ribosómica S6 (RPS6). Este ensayo se basa en el uso de microbolas paramagnéticas (6,5 μm de diámetro) para fijar los analitos y los sustratos de reacción, permitiendo así la cuantificación de múltiples analitos en un solo ensayo. Los ensayos se realizaron siguiendo las instrucciones del fabricante. Así, los patrones y las muestras se analizaron por duplicado, realizándose la incubación durante la noche, con agitado a 4 °C (18 horas, 750 rpm), y empleando un bloque magnético manual en los pasos de lavado. Los datos se obtuvieron mediante el sistema MagPix 200 de Luminex (Luminex, Molecular Diagnostics, Toronto, Canadá) y se analizaron con el programa XPonent (Luminex, Molecular Diagnostics, Toronto, Canadá).

Para el análisis de tejido hepático, se homogenizaron 100 mg de tejido hepático en un tampón de lisis (Tris-HCl 20 mM [pH 7.4] que contenía 1 % de Triton X-100, 0,1 % de dodecil sulfato de sodio, 50 mM de NaCl, 2.5 mM de ácido etilenodiaminotetracético, 1 mM de Na_4_P_2_O_7_·10H_2_O, 20 mM de NaF, 1 mM de Na_3_VO_4_, 2 mM de Pefabloc y Complete de Roche). Posteriormente, se cuantificó y normalizó la concentración total de proteínas en todas las muestras, con el kit de ensayo de proteínas BCA Pierce (Thermo Fisher Scientific, MA, EE.UU). Los resultados se expresan como mediana de intensidad de fluorescencia (MIF).

### Análisis *Western blot*: niveles de expresión de las proteínas Akt y FoxO1

La abundancia de proteínas Akt y FoxO1 en el tejido, así como su grado de fosforilzación se determinaron mediante *Western blot*. Los lisados de tejidos se prepararon en un tampón de lisis. A continuación, se separaron las proteínas en un gel de poliacrilamida con SDS al 10 % (Mini Protean III; Bio-Rad, Richmond, CA) y se transfirieron a membranas de nitrocelulosa de 0,45 µm durante 2 horas a 48 °C. Después del bloqueo, las membranas se incubaron a 48 °C durante la noche con los siguientes anticuerpos: anti-FoxO1 de conejo (1:1,000; Señalización celular), anti-Akt de conejo (1:5,000; Señalización celular), anti-fosfo-Akt de conejo (Ser473) (1:5,000; Señalización celular) y anticuerpo antitubulina (1:5,000 Señalización celular). Posteriormente, las membranas se incubaron con anticuerpos secundarios conjugados con perixidasa a una dilución de 1:5,000 dilution (GE Healthcare) durante una hora a temperatura ambiente. Las respectivas bandas se visualizaron empleando sustrato Luminata Forte Western HRP (Millipore) e Image-Quant LAS 4000 (GE Healthcare). Se realizó un análisis de densiometría de los geles con el programa ImageJ (versión 1.37). Los resultados se expresan en unidades densitométricas relativas (UDR) determinadas a partir de las relaciones densitométricas de p-Akt/Akt y FoxO1/tubulina. Con el fin de establecer la linealidad cuantitativa de los experimentos de *Western blot*, empleamos proteínas de tubulina recombinante y Akt 1 total recombinante a diferentes concentraciones (proteína tubulina humano-recombinante ab70187 y proteína AKT1 humano-recombinante-ab79792; Abcam).

### Análisis estadístico

Las variables continuas se expresan como mediana ± rango intercuartílico (RIC). Las diferencias en las variables cuantitativas se evaluaron mediante el test *U* de Mann-Whitney. El grado de correlación entre variables se determinó mediante el coeficiente de Pearson o de Spearman, según la distribución de la variable. Para el análisis de regresión lineal y correlación lineal, se transformó logarítmicamente la variable FoxO1 (LnFoxO1), para corregir la falta de simetría en la distribución. Los parámetros que mostraron una correlación significativa o casi significativa con los parámetros analíticos de daño y disfunción hepática fueron incluidos en el análisis de regresión múltiple. La técnica de análisis estadístico multivariante empleada fue la regresión lineal. La validez de los modelos resultantes se evaluó representando en una gráfica los residuos o residuos de Pearson frente a los valores ajustados por el modelo, y mediante una gráfica que mostraba la influencia de cada observación sobre la respuesta, ajustada en función de la distancia de Cook. Todos los análisis estadísticos se realizaron utilizando las bibliotecas públicas de la Comprehensive R Archive Network (CRAN; http://CRAN.R-project.org), en el entorno de cálculo estadístico de código abierto R, versión 3.1 (http://www.R-project.org/). Para la representaciones gráficas, se empleó el programa GraphPad Prism versión 8.0,2 (GraphPad Software, CA, USA). Un valor p<0,05 se consideró estadísticamente significativo. La revisión estadística del estudio la realizó un especialista en estadística biomédica.

## Resultados

### Parámetros analíticos de daño y función hepática

En la Tabla 1 se muestran las características demográficas de los sujetos de estudio. Aunque algunos estudios sugieren que FoxO1 podría estar implicada en las patologías asociadas a la edad y a la esperanza de vida, no hallamos diferencias significativas entre los grupos, en la edad de los sujetos (p=0,651).

Todos los pacientes cirróticos presentaron valores elevados de aminotransferasas (ALAT y ASAT) y GGT, indicativas de daño hepático (Tabla 2). Estas elevaciones en los marcadores bioquímicos únicamente se observaron en las muestras séricas de pacientes cirróticos.

**Tabla 2:** Parámetros analíticos determinados en muestras de sangre de pacientes cirróticos.

		No cirróticos (N=9)		Cirróticos (n=27)		
Parámetro	Intervalo de referencia	N	Mediana (RIC)	n	Mediana (RIC)	Valor p
ASAT	5–40 IU/L	8	22,5 (18,5–24,0)	25	71,0 (52,0–114,0)	<0,001
ALAT	5–40 IU/L	8	20,0 (15,5–23,0)	25	113,0 (72,0–165,0)	<0,001
GGT	5–40 IU/L	7	28,0 (14,0–45,0)	25	71,0 (38,0–140,0)	0,03
Albúmina	34–48 g/L	7	43,0 (32,0–46,0)	24	43,0 (39,5–45,0)	NS
Bilirrubina	<1,2 mg/dL	8	0,8 (0,60–1,0)	22	0,8 (0,6–1,0)	NS
Recuento de plaquetas	130–400 × 10^9^/L	8	236,5 (206,0–286,5)	25	128 (99,0–162,0)	<0,001
Tiempo de protrombina	80–100 %	8	86,0 (76,0–97,5)	25	92,0 (86,0–100,0)	NE
Glucosa	65–110 mg/dL	8	95,5 (88,0–120,0)	25	99,0 (87,0–119,0)	NE
Colesterol	≤200 mg/dL	8	190,5 (176,0–207,5)	24	164,5 (149,5–180,5)	0,05
Triglicéridos	<150 mg/dL	8	93,5 (66,0–102,5)	24	99,5 (86,5–135,0)	NS
Alfafetoproteína	<10 ng/mL	N.D.	N.D	20	12,5 (7,5–49,0)	N.D

Las variables se expresan como mediana ± rango intercuartílico. UI, unidades internacionales; ALAT, alanina aminotransferasa; ASAT, aspartato aminotransferasa; GGT, gamma-glutamil transferasa; N, número total de pacientes en cada grupo; n, número de pacientes en los que se pudo determinar el parámetro; RIC, rango intercuartílico; N.D., no determinado.

### Estudio comparativo: expresión intrahepática de las proteínas de la vía Akt en pacientes cirróticos

**Figura 1:**
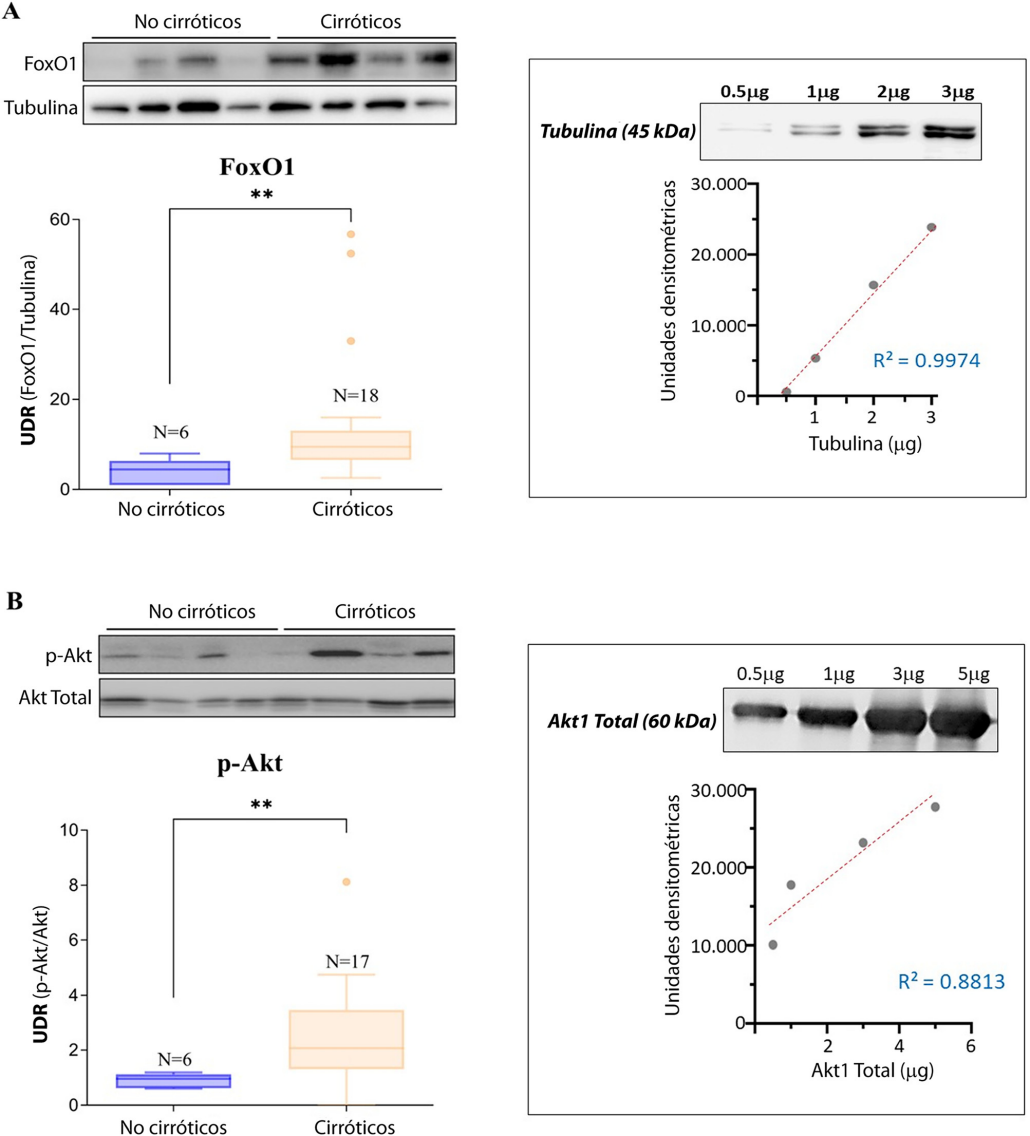
Expresión de las proteínas FoxO1 y p-Akt en el hígado de los pacientes cirróticos. Diagrama de cajas comparando los niveles de FoxO1 (A) y p-Akt (B), expresados en unidades densiométricas relativas (UDR). La UDR se calculó como la proporción entre las bandas específicas de FoxO1/tubulina y p-Akt/Akt, cuantificadas con el programa ImageJ. En la parte superior de cada figura se muestra una imagen representativa de los análisis mediante *Western blot*. Se observaron diferencias estadísticamente significativas (**p<0,01) al comparar la mediana de las dos proteínas de los dos grupos: FoxO1: mediana=4.4 (no cirróticos) frente a 9.5 (cirróticos), p-Akt: mediana=1.0 (no cirróticos) frente a 2.1 (cirróticos). Las cajas de la parte derecha de los paneles A y B muestran la capacidad cuantitativa de los *Western blots* demostrando la linealidad entre las unidades densiométricas medidas y las concentraciones elevadas de proteínas recombinantes para tubilina y Akt1 total, que se seleccionaron como controles de carga. Las líneas de regresión obtenidas a partir de las proteínas recombinantes mostraron un R^2^ aceptable.

Con respecto a los niveles de expresión de FoxO1 y Akt en tejido hepático, los resultados muestran niveles aumentados de p-Akt (forma activa) en los pacientes cirróticos, con diferencias estadísticamente significativas (p<0,01; véase la Figura 1). No se hallaron diferencias estadísticamente significativas en los niveles de Akt entre los grupos. En la Figura 1B se muestran los niveles de FoxO1, regulados negativamente por Akt. La mediana de los niveles de FoxO1 fue significativamente superior en los pacientes cirróticos, frente a los no cirróticos (p<0,01).

Entre las proteínas diana analizadas por Luminex^®^ (GSK3β, IGF1R, IRS1, mTOR, p70S6K, IR, PTEN, GSK3α, TSC2 y RPS6), se observaron diferencias estadísticamente significativas en dos proteínas: p70S6K y PTEN.

**Figura 2:**
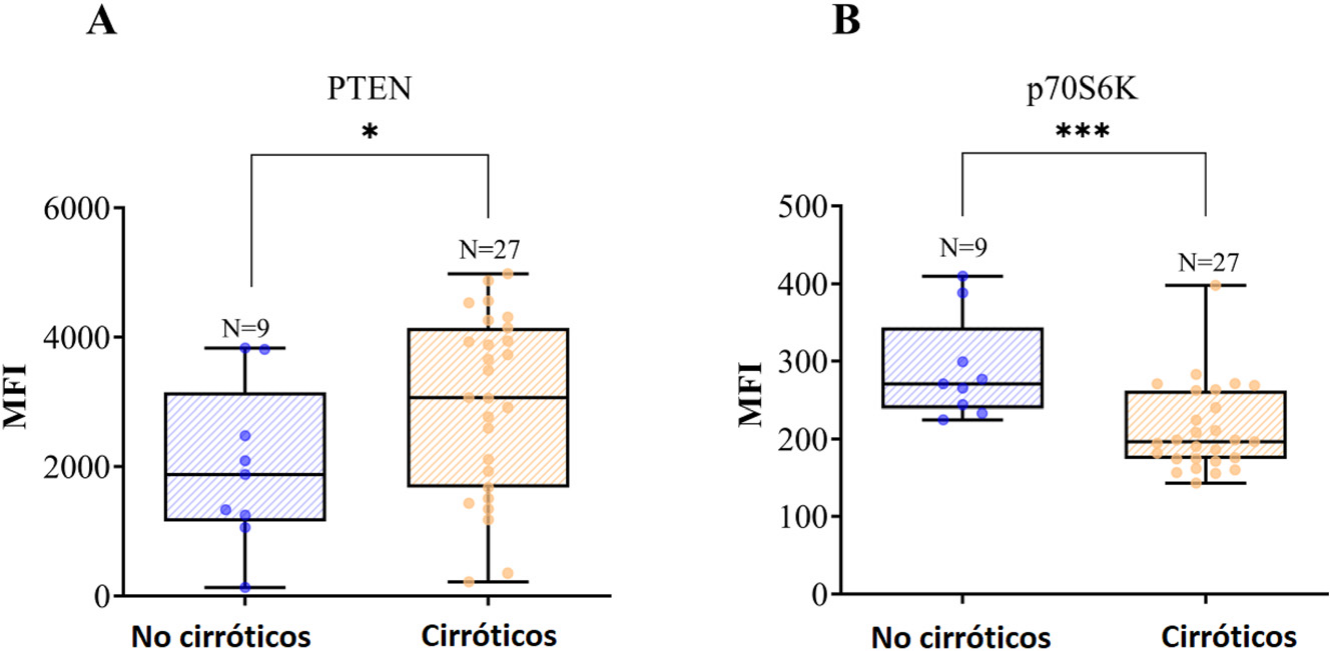
Diagrama de cajas en el que se representan los niveles de PTEN (A) p70S6K (B) en los dos grupos. Los niveles de cada proteína en el tejido hepático se expresan como IMF (intensidad media de fluorescencia). A: Los niveles de PTEN fueron significativamente inferiores en los pacientes no cirróticos (mediana=1,877, n=9) comparados con los pacientes con cirrosis (mediana=3,061, n=27) (*p<0,05). B: Los pacientes no cirróticos mostraron niveles significativamente superiores de p70S6K (270,5; n=9) comparados con los pacientes cirróticos (196,3; n=27) (***p<0,001).

Tal como muestra la Figura 2, la mediana de los niveles de PTEN fue significativamente superior en el grupo de cirróticos (p<0,05). Además, los pacientes con cirrosis mostraron niveles considerablemente inferiores de p70S6K (p<0,01). No hubo diferencias significativas en el resto de variables (Tabla 3).

**Tabla 3:** Estudio comparativo de 12 proteínas diana en la vía Akt.

	No cirróticos (N=9)		Cirróticos (n=27)		
Parámetro	N	Mediana (RIC)	n	Mediana (RIC)	Valor p
^a^FoxO1	6	4,4 (1,0 – 6,4)	18	9,5 (6,6 – 13,0)	<0,01
p-Akt	6	1,0 (0,6 – 1,1)	17	2,1 (1,3 – 3,5)	<0,01
+GSK3α	9	123,0 (25,5 – 144,8)	27	158,5 (102,8 – 232,3)	NS
GSK3β	9	73,5 (66,8–155)	27	83,5 (64,0 – 112,5)	NS
IGF1R	9	2,5 (0 – 6,5)	27	5,0 (0 – 10,5)	NS
IR	9	8,5 (0 – 11,0)	27	9,0 (3,8 – 14,5)	NS
IRS1	9	57,5 (32,5 – 73,5)	27	66,8 (44,5 – 96,5)	NS
mTOR	9	43,5 (34,5 – 60,3)	27	52,0 (36,3 – 83,0)	NS
p70S6K	9	270,5 (243,8 – 299,0)	27	196,3 (174,9 – 262,0)	<0,01
PTEN	9	1876,5 (1,245,0 – 2,479,8)	27	3060,8 (1,676,5 – 4,142,3)	0,04
RPS6	9	103,3 (64,0 – 127,5)	27	97,3 (44,5 – 268,5)	NS
TSC2	9	105,5 (49,5 – 193,5)	27	162,5 (105,3 – 260,0)	NS

Las diferencias entre grupos se evaluaron con el test *U* de Mann-Whitney. Un valor p<0,05 se consideró estadísticamente significativo. ^a^Los valores de FoxO1 y p-Akt se expresan en unidades densiométricas relativas (UDR), calculadas como la relación densiométrica entre FoxO1/tubulina y p-Akt/Akt. +Las proteínas restantes se expresan como IMF (intensidad media de fluorescencia). RIC, rango intercuartílico; N, número total de pacientes en cada grupo; n, número de pacientes en los que se pudo determinar el parámetro.

### Correlación entre las proteínas de la vía Akt y los parámetros analíticos de daño y función hepática

**Figura 3:**
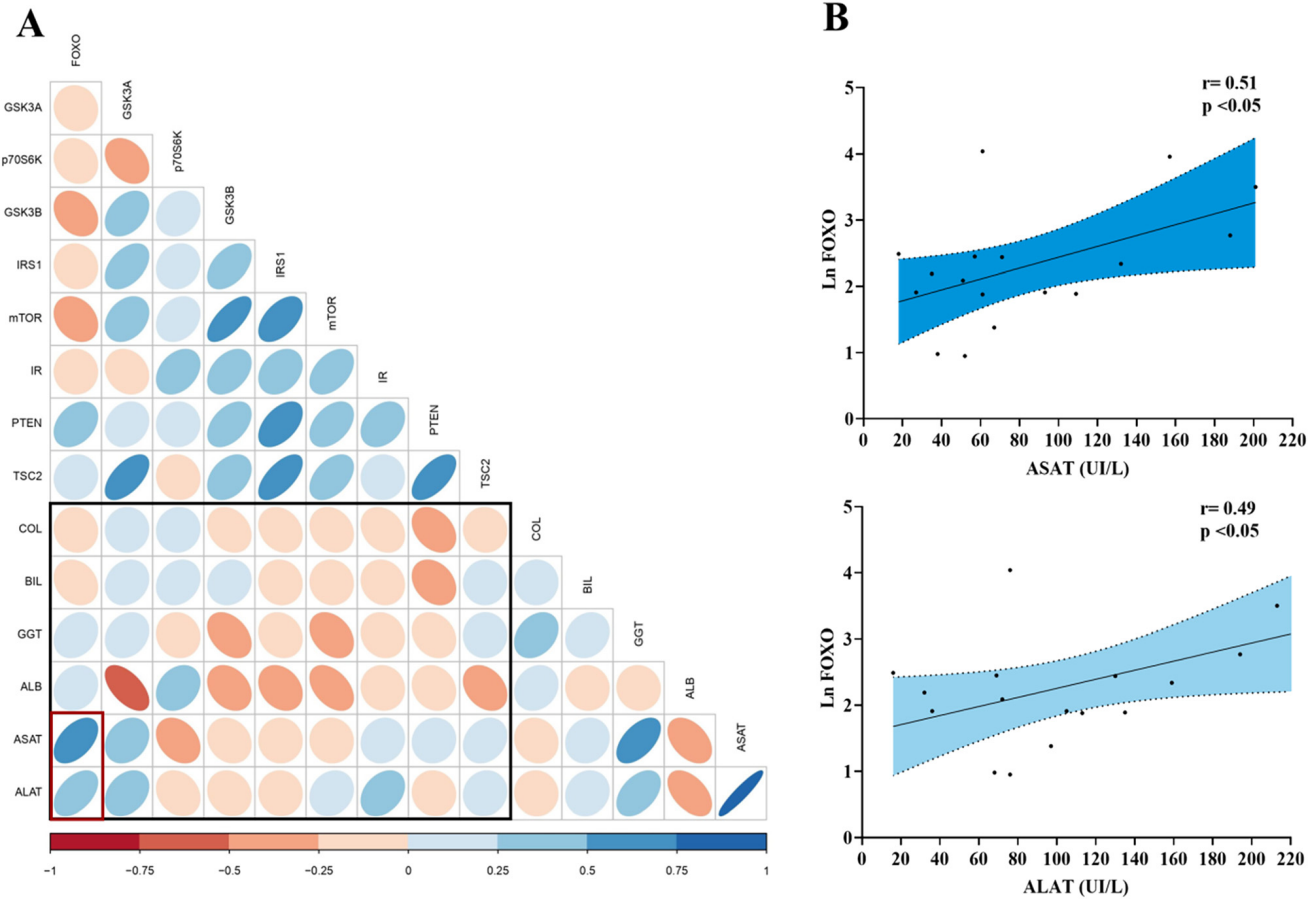
Matriz de correlación entre las proteínas de la vía Akt y los parámetros analíticos de daño y función hepática. (A) Se muestran todas las posibles correlaciones entre las variables estudiadas. El rectángulo negro señala las correlaciones de interés: las correlaciones más relevantes entre FoxO1 y las transaminasas están indicadas con un recuadro rojo. La escala de color indica si la correlación es positiva (en azul) o negativa (en rojo). La intensidad del color y el tamaño de las elipses son proporcionales a los coeficientes de correlación (Pearson). (B) El diagrama de dispersión indica que existe una correlación positiva entre los niveles de proteína FoxO1 en tejido hepático y la concentración de transaminasas (ASAT y ALAT) en suero. Estas correlaciones fueron estadísticamente significativas, con los siguientes coeficientes de Pearson: r=0,51 para ASAT y r=0,49 para ALAT. También se representa la línea de regresión correspondiente; el área sombreada indica los intervalos de confianza al 95 %.

Se analizó la asociación entre las proteínas de Akt y los parámetros analíticos de función y daño hepático. En la Figura 3 se muestra la matriz de correlación resultante de dicho análisis. Los grados de correlación lineal, así como la significación, se calcularon mediante los coeficientes de correlación de Pearson. Las variables que no seguían una distribución normal se excluyeron de dicha matriz, y sus relaciones se evaluaron individualmente mediante el coeficiente de correlación de Spearman. Observamos una correlación negativa entre la expresión de GSK3α y los niveles de albúmina en suero (r=−0,59, p=0,002), y una correlación positiva con los niveles de ASAT (r=0,42; p=0,039). FoxO1 fue la proteína que mostró mayor correlación con las pruebas de daño hepático. Los niveles de expresión de la proteína FoxO1 y las concentraciones séricas de ambas transaminasas mostraron una correlación positiva (moderada a alta) estadísticamente significativa. ASAT (r=0,51, p=0,036) y ALAT (r=0,49, p=0,049). La correlación lineal y la línea de regresión ajustada de FoxO1 correspondiente y las transaminasas se detalla en la Figura 3B. La expresión de FoxO1 mostró una correlación positiva moderada con la puntuación FIB-4 (r=0,46, p=0,036).

### El análisis de regresión explica la influencia de FoxO1 en los marcadores bioquímicos de daño hepático

Dada la correlación estadísticamente significativa observada entre las transaminasas en suero y las proteínas FoxO1 y GSK3α, las dos dianas de Akt se incluyeron en el análisis multivariante, que reveló que los dos parámetros eran predictores positivos independientes de los niveles de ASAT, explicando el 37 % de la varianza (R^2^-adj=0,37; F=5.75; p=0,015). En el análisis de regresión lineal multivariante de la variable dependiente ALAT, únicamente se identificó a FoxO1 como predictor positivo independiente, explicando el 18 % de la varianza (R^2^-adj=0,18; F=4,61; p=0,049) (Tabla Suplementaria 1).

## Discusión

La vía de señalización PI3K/AKT es crucial para la homeostasis energética, el crecimiento celular y la supervivencia. Los estudios en roedores demuestran el papel fundamental de Akt en la regeneración hepática, así como en la función hepática normal [10]. Las acciones de la vía Akt en el ciclo celular están estrechamente relacionadas con la inhibición de FoxO1, un miembro de la familia de factores de transcripción FoxO. Estos factores de transcripción son necesarios para múltiples funciones hepáticas: regulan la respuesta al estrés, la adaptación al ayuno, y la proliferación celular [11]. Akt inactiva la proteína FoxO1 mediante fosforilación, lo que deriva en su exclusión nuclear, inhibiendo así su señalización proapoptótica [12]. Por esta razón, la interacción Akt-FoxO1 se ha identificado como el mecanismo más importante de regeneración hepática. En resumen, la preservación de la actividad Akt y la consiguiente inactivación de FoxO1 están asociadas a una mejor funcionalidad y capacidad de regeneración del hígado. De este modo, Akt y FoxO1 se postulan como prometedoras dianas terapéuticas para la cirrosis y otras patologías hepáticas crónicas. Sin embargo, actualmente no existe evidencia suficiente del nivel de activación de estas moléculas en los pacientes con cirrosis, lo que impide su posible traslado a la clínica.

Este es el primer estudio en evaluar de forma detallada el estado de activación de las moléculas de la vía de señalización Akt en el hígado cirrótico. En nuestros estudios en modelos experimentales de ratas cirróticas, demostramos que la actividad enzimática de Akt disminuía en los hígados disfuncionales [2]. Sin embargo, en el presente estudio en pacientes cirróticos tras una hepatectomía, observamos que Akt y su diana FoxO1 estaban sobreexpresadas en los hígados cirróticos. Esta discrepancia debería ser interpretada con cautela, dado que se puede atribuir a otros factores, como las comorbilidades de los pacientes cirróticos o la etiología de la enfermedad. Esta última no fue un factor relevante en el modelo animal, donde se indujo la cirrosis mediante la inhalación de tetracloruro. En nuestra cohorte de pacientes con cirrosis, la etiología basal en todos los casos fue infección por el virus de la hepatitis C. Aún se desconoce la relación entre la vía Akt y los procesos de replicación viral en infección crónica por VHC. Los estudios *in vitro* sugieren que la proteína viral NS5A puede activar la vía PI3K-Akt, causando así la inhibición de la apóptosis celular [13]. Algunos autores han descrito esta relación como un mecanismo evolutivo de la mayoría de los virus de ADN de mamíferos, que promueve la supervivencia celular y garantiza su replicación [14].

Los resultados de este estudio subrayan la importancia de validar en humanos los resultados preclínicos obtenidos para las moléculas implicadas en la vía Akt. El hallazgo más relevante es que FoxO1 está sobreexpresado en los hígados con cirrosis. Este hallazgo concuerda con estudios anteriores, que demuestran una sobreexpresión de FoxO1 en el tejido hepático de pacientes con otra patología hepática crónica de diferente etiología, la esteatohepatitis no alcohólica [15].

La sobreexpresión de FoxO1 es clínicamente relevante, ya que la mayoría de los pacientes diagnosticados de HCC presentan cirrosis subyacente [16]. Actualmente, la resección quirúrgica de estos tumores o lesiones metastásicas en pacientes con cirrosis sigue siendo compleja, debido a su morbimortalidad [17]. En pacientes con cirrosis, la capacidad de regeneración de los hepatocitos tras la cirugía es reducida, lo que limita la aplicabilidad de la resección terapéutica en contextos como el HCC [18]. Esta observación refuerza la hipótesis de que, cuanto mayor sea la expresión de FoxO1, mejor será la capacidad de regeneración del hígado, tal como se ha demostrado en un modelo murino de regeneración hepática causada por una hepatectomía parcial [8]. Además, este hallazgo es consistente con la correlación significativa observada en nuestro estudio entre los niveles de FoxO1 y el grado de daño hepático. Nuestro modelo estadístico reveló que, de las moléculas estudiadas, FoxO1 es la única en haber demostrado ser un factor predictor independiente de los niveles de ALAT y ASAT en suero. Teniendo en cuenta estos resultados, así como los obtenidos en estudios preclínicos, la sobreexpresión de FoxO1 podría ser uno de los mecanismos subyacentes que limitan la regeneración del tejido cirrótico.

La principal limitación de nuestro estudio es su tamaño muestral, por lo que estos resultados preliminares precisan ser validados en estudios prospectivos multicéntricos, en una muestra mayor. Es importante confirmar esta hipótesis, ya que los niveles intrahepáticos de FoxO1 podrían ser un indicador de la capacidad de regeneración hepática, siendo por tanto un marcador con valor pronóstico en pacientes con cirrosis y HCC. Es necesario dilucidar los mecanismos de inducción y regulación de la regeneración hepática, para poder identificar nuevas dianas terapéuticas. Esto permitiría desarrollar terapias basadas en el restablecimiento de la funcionalidad hepática, mejorando así el pronóstico de los pacientes con patología hepática avanzada.

Nuestros resultados muestran la necesidad de darle continuidad a esta línea de investigación, para conocer mejor la implicación de la vía Akt-FoxO1 en la cirrosis. La estrategia ideal sería realizar estudios observacionales prospectivos, con el fin de determinar el estado de esta vía en la población con cirrosis de diferentes etiologías. Como futura línea de investigación, resultaría útil investigar otros métodos menos invasivos que la biopsia hepática, para estudiar la expresión de esta vía. Concretamente, la biopsia líquida, como el aislamiento de exosomas circulantes procedentes de diversos fluidos biológicos, unido al análisis de sus contenidos (esto es, el ARN, proteínas, y lípidos), se postulan como una estrategia prometedora para la caracterización de los perfiles genómicos y transcriptómicos de las enfermedades hepáticas. En futuros estudios se podrá obtener evidencia que respalde la teoría de que la modulación selectiva de la vía Akt o de sus dianas es una estrategia viable con efectos beneficiosos en el manejo de pacientes con patologías hepáticas.

## Supplementary Material

Supplementary Material

## Material Suplementario

Este artículo incluye material suplementario (https://doi.org/10.1515/almed-2023-0054).
