# Supplementary material for: La sobreexpresión de FoxO1 en el hígado esta positivamente asociada al grado de daño hepático en pacientes cirróticos
Source: Adv Lab Med. 2023 Aug 4;4(3):227–35. [Article in Spanish] doi: 10.1515/almed-2023-0054 (PMC10701490; doi:10.1515/almed-2023-0054)
Supplement: Supplementary file 1 — Supplementary Material [file j_almed-2023-0054_suppl_001.docx]

MATERIAL SUPLEMENTARIO

**Tabla 1.** Resultados del análisis de regresión múltiple.

| Variable dependiente  ASAT | F(2,14)=5,75 R^2^=0,45 R^2^ -adj=0,37 Valor *p*=0,015 | | | | |
| --- | --- | --- | --- | --- | --- |
|  | Coef. | Err. Est. | β | Valor *p* | Intervalo de confianza al 95% |
| Constante | -52,98 | 41,72 |  |  | -142,46 ̶ 36,51 |
| FoxO1 | 34,07 | 12,41 | 0,54 | 0,016 | 7,47 ̶ 60,69 |
| GSK3A | 0,38 | 0,17 | 0,44 | 0,045 | 0,01 ̶ 0,75 |
| Variable dependiente  ALAT | F(1.15)=4.61 R^2^=0.24 R^2^-adj=0.18 p-Value=0.049 | | | | |
|  | Coef. | Err. Est. | β | Valor *p* | Intervalo de confianza al 95% |
| Constante | 28,20 | 39,25 |  |  | -55,45 ̶ 111,86 |
| FoxO1 | 34,20 | 15,96 | 0,49 | 0,049 | 0,25 ̶ 68,30 |

ASAT: aspartato aminotransferasa, ALAT: alanina aminotransferasa.
